# Antiviral Activity of Ribosome-Inactivating Proteins

**DOI:** 10.3390/toxins13020080

**Published:** 2021-01-22

**Authors:** Lucía Citores, Rosario Iglesias, José M. Ferreras

**Affiliations:** Department of Biochemistry and Molecular Biology and Physiology, Faculty of Sciences, University of Valladolid, E-47011 Valladolid, Spain; luciac@bio.uva.es (L.C.); riglesia@bio.uva.es (R.I.)

**Keywords:** adenine polynucleotide glycosylase, antiviral therapy, human virus, immunotoxin, ribosome-inactivating protein (RIP), rRNA glycosylase (EC 3.2.2.22), virus-resistant transgenic plant (VRTP)

## Abstract

Ribosome-inactivating proteins (RIPs) are rRNA N-glycosylases from plants (EC 3.2.2.22) that inactivate ribosomes thus inhibiting protein synthesis. The antiviral properties of RIPs have been investigated for more than four decades. However, interest in these proteins is rising due to the emergence of infectious diseases caused by new viruses and the difficulty in treating viral infections. On the other hand, there is a growing need to control crop diseases without resorting to the use of phytosanitary products which are very harmful to the environment and in this respect, RIPs have been shown as a promising tool that can be used to obtain transgenic plants resistant to viruses. The way in which RIPs exert their antiviral effect continues to be the subject of intense research and several mechanisms of action have been proposed. The purpose of this review is to examine the research studies that deal with this matter, placing special emphasis on the most recent findings.

## 1. Introduction

One of the main efforts of virologists and molecular biologists is the search for antivirals that can help in the fight against viruses causing diseases in animals and especially in humans. Strategies are also being searched to tackle the challenge of plant viruses causing significant crop losses. This has led to the discovery of a number of antivirals with different chemical nature or proteins with different enzymatic activities [1,2]. The search for more effective and safer antivirals continues to be a field of intense investigation and plants are one of the most used sources, since they have evolved a variety of protein-based defense mechanisms to tackle viral infections [3]. Regarding ribosome-inactivating proteins (RIPs), it is worth noting the fact that one of the first RIPs to be purified was PAP (pokeweed antiviral protein) and although many RIPs have been purified as protein synthesis inhibitors, many others have been isolated as powerful antivirals. For many years, RIPs have been studied as potent inhibitors of protein synthesis that can be used for the construction of immunotoxins [4]. Since linked to a monoclonal antibody or a protein that specifically binds to a receptor, they can be used to specifically kill tumor cells [4,5]. RIPs have initially been studied as a family of proteins widely distributed among angiosperms although they have also been found in other taxons [6,7]. They irreversibly inactivate ribosomes inhibiting protein synthesis and thus causing cell death [6,7]. The first RIPs to be isolated, the extremely potent toxins ricin and abrin, were purified at the end of the nineteenth century and it was believed that their red cell agglutinating activity was the reason for the toxic effect [8,9]. In the early 1970s, it was reported that abrin, ricin, and PAP strongly inhibited protein synthesis in a cell-free rabbit reticulocyte system [8,9,10]; and Barbieri and Stirpe classified these and other related proteins as type 1 RIPs (a single polypeptide chain, such as PAP) and type 2 RIPs (two chains, an A chain similar to type 1 RIPs, and a B chain that possesses lectin activity, such as abrin and ricin) [4]. The enzymatic activity of ricin was discovered by Endo and colleagues, that is, RIPs are considered as 28S rRNA N-glycosylases (EC 3.2.2.22) that cleave the N-glycosidic bond between the adenine No. 4324 and its ribose in the 60S subunit of rat ribosomes [11] or the equivalent one in sensitive ribosomes from other organisms [12]. This adenine is located in the sarcin-ricin loop (SRL) that is crucial for anchoring the elongation factors EFG and EF2 on the ribosome during mRNA-tRNA translocation in prokaryotes and eukaryotes, respectively. This loop is also the target of ribotoxins such as α-sarcin, enzymes with rRNA endonuclease activity (EC 3.1.27.10) [13]. However, some RIPs are also able to remove more than an adenine from the rRNA [14] and many of them are able to deadenylate not only rRNA but also other polynucleotide substrates such as DNA, poly(A), mRNA, tRNA, and viral RNA [15], and because of this, the name of adenine polynucleotide glycosylase (or polynucleotide: adenosine glycosidase) was proposed for RIPs [15]. Additionally, other activities have been reported for RIPs, just as shown in Table 1.

A convincing picture of the role played by these proteins in plants is not yet available. They seem to play different roles in different species, so antiviral, antifungal, plant defense, storage, programmed senescence, antifeedant, stress protection, and development regulation roles have been proposed for RIPs [7].

The need to find new antivirals has encouraged researchers to study the antiviral activity of RIPs. On the other hand, much research is underway, focused on the use of these proteins to obtain crops with resistance to viral pathogens. The aim of this review is to compile the advances that have been made within this field, placing special emphasis on the most recent findings.

## 2. Activity on Animal (Human) Viruses

Global health threats such as the emergence of human viruses resistant to commonly used antiviral drugs, has prompted the study of RIPs as possible tools for fighting these agents. Antiviral activity of RIPs against different animal viruses has been reported (Table 2).

RIPs with antiviral activity belong to the main types of RIPs found in angiosperms [7]: monocot type 1 RIPs (Poaceae), dicot type 1 RIPs (Euphorbiaceae, Caryophyllaceae, Phytolaccaceae), type 2 RIPs (ricin, Euphorbiaceae), and type 1 RIPs derived from type 2 RIPs (Cucurbitaceae); which suggests that all these proteins could have, to a greater or lesser extent, antiviral activity and that their main biological role could be precisely the defense of the plant against viruses. However, researchers have focused on the study of proteins obtained from species of the families Phytolaccaceae, Cucurbitaceae, Caryophyllaceae, and Euphorbiaceae; and the most studied RIPs are pokeweed antiviral protein (PAP), trichosanthin (TCS) and Momordica antiviral protein (MAP30), which have been the subject of recent reviews [10,35,36,38,58]. It is noteworthy that RIPs have shown to be active against viruses of very different nature: double-stranded (ds) DNA viruses (hepatitis B virus, HBV; human gammaherpesvirus, HHV; human poliovirus, HPV; herpes simplex virus, HSV), retroviruses (human immunodeficiency virus, HIV; human T-cell leukemia virus, HTLV; simian–human immunodeficiency virus, SHIV), positive-sense single-stranded (ss) RNA viruses (Japanese encephalitis virus, JEV; dengue virus, DENV; chikungunya virus, CHIKV), and negative-sense (ss) RNA viruses (human influenza virus, FLUV; lymphocytic choriomeningitis virus, LCMV; Pichinde virus, PICV). Most of the viruses studied are enveloped viruses that infect humans, with the exceptions of the simian–human immunodeficiency virus (SHIV), the Pichinde virus (PICV), and the non-enveloped human poliovirus. This virus was the first in which activity against an animal virus was reported [59]. Results obtained with HEp-2 cells infected with human poliovirus or herpes simplex virus (HSV) showed that gelonin, momordin, dianthin 32, and PAP-S impaired viral replication by inhibiting protein synthesis in virus-infected cells, in which presumably they enter more easily than in uninfected cells [30], suggesting that antiviral activity could be a general property of RIPs.

### 2.1. Activity on Human Immunodeficiency Virus

The most studied virus is the human immunodeficiency virus (HIV). The lack of effective antivirals against this virus and its rapid spread around the world prompted studies on the activity of RIPs against this virus since 1989 [60]. At least 20 RIPs have shown activity against HIV (Table 2). Thus, several RIPs obtained from Euphorbiaceae and Caryophylaceae, but mostly from Cucurbitaceae and Phytolocaceae, inhibit the replication of HIV in vitro [35]. It has also been reported that maize RIP transiently reduces viral load in SHIV infected Chinese rhesus macaques [27]. The results obtained with RIPs promoted their use in clinical trials [61]. Although the development of specific HIV antivirals such as reverse-transcriptase and protease inhibitors have directed AIDS therapy to other treatments, these studies demonstrated the potential of RIPs for the treatment of virus-related diseases.

### 2.2. Activity on Herpes Simplex Virus

Another virus that has been targeted by RIPs is the herpes simplex virus (HSV). Currently, there is no treatment that completely eliminates HSV infection from the body, because once the virus enters an organism, it remains dormant until reactivated. This has encouraged researchers to study RIPs as candidates for HSV therapy. Gelonin, trichosanthin, dianthin 32, PAP, PAP-S, and several RIPs obtained from *Momordica charantia* have shown anti-HSV activity in vitro (Table 2).

### 2.3. Activity on Other Animal Viruses

Exposure of HepG2.2.15 cells to MAP30 [44], PAP-S [56], α-momorcharin [41], and an eukaryotic expression plasmid encoding PAP [56] inhibits the production of hepatitis B virus (HBV). Additionally, an extract from Radix Trichosanthis had a stronger inhibitive effect on expression of HBsAg and HBeAg in HepG2.2.15, and trichosanthin has been proposed as the main component of the aqueous extract responsible for the anti-hepatitis B viral effect [62].

On the other hand, it has also been reported that PAP inhibits replication of human T-cell leukemia (HTLV), human influenza, chikungunya (CHIKV), Japanese encephalitis (JEV), and lymphocytic choriomeningitis (LCMV) viruses, gelonin inhibits Pichinde virus replication, and MAP30 inhibits human gammaherpesvirus 8 (HHV8) and dengue virus [10,31,35,42,52,53,54,55].

### 2.4. Citotoxicity of RIPs

An important aspect to consider when working with antivirals is their cytotoxicity. In this sense, type 1 RIPs and type 2 RIPs can be distinguished. Type 1 RIPs consist of a polypeptide chain with rRNA N-glycosylase activity, while type 2 RIPs are constituted by two chains linked by a disulfide bond: The A chain (active) is equivalent to a type 1 RIP and the B chain (binding) is a lectin able to bind to membrane glycoproteins and glycolipids allowing endocytosis of RIP by cells. This is why RIPs such as ricin and abrin are extremely toxic showing IC_50_ (concentration that inhibits protein synthesis by 50%) values of 0.67–8 pM in cell cultures [63]. There are type 2 RIPs such as those from *Sambucus* which are much less toxic to cultured cells with IC_50_ values of 27–64 nM [64]. Type 1 RIPs are much less toxic and have highly variable IC_50_ values (0.2–10 μM) [63]. Due to the high cytotoxicity of type 2 RIPs, only type 1 RIPs or the ricin A-chain (which has a cytotoxicity similar to that of type 1 RIPs) [63] have been used as antiviral agents.

A good antiviral should display a substantial difference between the antiviral concentration and the cytotoxic concentration. Due to the diverse toxicities of type 1 RIPs, there are also differences in this regard, but the most commonly used proteins such as PAP, MAP30, or trichosanthin always show a substantial difference between toxic concentrations for cells (3–30 μM) [63,65,66] and concentrations that have antiviral activity (around 30 nM) [35].

Finally, it should be noted that some bacterial and fungal enzymes targeting the sarcin-ricin loop have also been reported to possess antiviral activity [2,67,68,69,70,71,72,73].

Therefore, RIPs have awakened over many years, and continue to do so, a keen interest as tools to fight viruses that cause diseases in humans. In fact, recently saporin and RTAM-PAP1 (a chimera constructed with ricin A-chain and PAP) have been proposed as candidates for therapy of COVID-19 [74,75].

## 3. Activity against Plant Viruses

To date, 39 RIPs have been described that display some type of activity against plant viruses (Table 3).

These RIPs have been found in 26 plant species belonging to one family of monocotyledons and ten families of dicotyledons, that are distributed throughout the phylogenetic tree of angiosperms in a similar way to the RIP-containing plants [7], thus suggesting that most RIPs could be active against plant viruses. As a matter of fact, only two type 2 RIPs from *Sambucus nigra* (SNAI and SNLRP) have been reported to fail to protect transgenic plants against viral infection [76].

Despite the fact that these antiviral proteins are distributed in a great variety of families, most of them (thirty one) belong to the orders Caryophyllales and Lamiales (families Caryophyllaceae, Amaranthaceae, Phytolaccaceae, Nyctaginaceae, Basellaceae, Lamiaceae), which are RIPs with well-defined structural and phylogenetic characteristics [7].

RIPs seem to be active against a wide range of viruses (Table 3), all of them belonging to different families of positive-sense single-stranded (ss) RNA viruses. The exception is the geminivirus ACMV (African cassava mosaic virus), which contains a single-stranded circular DNA genome. They seem to protect all kinds of plants and, although the most commonly used plant for testing has been *Nicotiana tabacum* L., RIPs have also shown ability to protect other species of the genus *Nicotiana* (*N. benthamiana* Domin and *N. glutinosa* L.) as well as other species commonly used in research or crops such as *Brassica rapa* L. (=*B. parachinensis* L.H.Bailey) (choy sum), *Cyamopsis tetragonoloba* (L.) Taub. (guar), *Crotalaria juncea* L. (sunn hemp). *Phaseolus vulgaris* L. (common bean), *Momordica charantia* L. (bitter melon), *Beta vulgaris* L. (sugar beet), *Cucurbita pepo* L. (squash), *Solanum tuberosum* L. (potato), *Carica papaya* L. (papaya), *Chenopodium quinoa* Willd. (quinoa), or *Lycopersicon esculentum* Mill. (tomato).

It is difficult to compare the antiviral activity of the different RIPs because different criteria have been used to evaluate their antiviral capacity. In some cases, the putative antiviral character is based on their N-glycosylase activity on the virus genome [105]; all RIPs are able to release adenines from any kind of RNA or DNA, including viral genomes [4]. This adenine polynucleotide glycosylase activity has been detected by electrophoresis [87], or HPLC [103,105]. In many cases, the test has involved applying a RIP solution on the leaf surface of the plant together with the virus and comparing the result with the control that does not contain RIP. In some cases, the virus is applied simultaneously [86,92,113] and in others, sometime after the application of the RIP [90,115]. The evaluation of antiviral activity has been done by counting the number of lesions [88,93], the time of onset of symptoms [77,79], the number of infected plants [105], or the severity of the infection symptoms [78,115]. Virus levels have also been estimated by ELISA [99], Western blotting analysis [81], RT-PCR analysis [101], quantitative real-time PCR analysis [81,82], electron microscopy [92], or by determining the infection capacity of an extract from the infected plant [92]. Another approach has been the construction of virus-resistant transgenic plants [80,102]. The virus has been inoculated mechanically or by aphids [102] and the resistance has been determined by one of the methods listed above.

Other studies link RIPs to the defense of plants against viruses, especially studies of induction of RIPs through signaling compounds such as salicylic acid, hydrogen peroxide, or jasmonic acid, which are involved in the systemic acquired resistance (SAR) of plants against viruses and other pathogens. Thus, it has been reported that artichoke mottled crinkle virus (AMCV), salicylic acid, and hydrogen peroxide induce the expression of BE27 in both treated and untreated leaves of sugar beet plant [86,117]. On the other hand, it has been reported that alpha-momorcharin induces the generation of salicylic acid, jasmonic acid, and reactive oxygen species, which improve tobacco mosaic virus (TMV) tolerance [118]. Additionally, alpha-momorcharin induces the expression of the N gene [118], which encodes the N protein that recognizes the TMV replicase fragment and triggers signal transduction cascades, initiating a hypersensitive response (HR) and inhibiting the spread of TMV [118]. Other RIPs in which some type of elicitor activity has been reported are pokeweed antiviral protein II (PAPII) [104], CIP-29 [111], and CA-SRI [113,115]. By contrast, the antiviral activity of SNAI’ [116], IRIP and IRAb [77], and nigrin b [76] is not accompanied by an induction of pathogenesis-related proteins. All this suggests that some, but not all RIPs, could be part of the SAR or/and HR to defend the plant against viral infections.

## 4. Antiviral Mechanisms of RIPs

**Figure 1:**
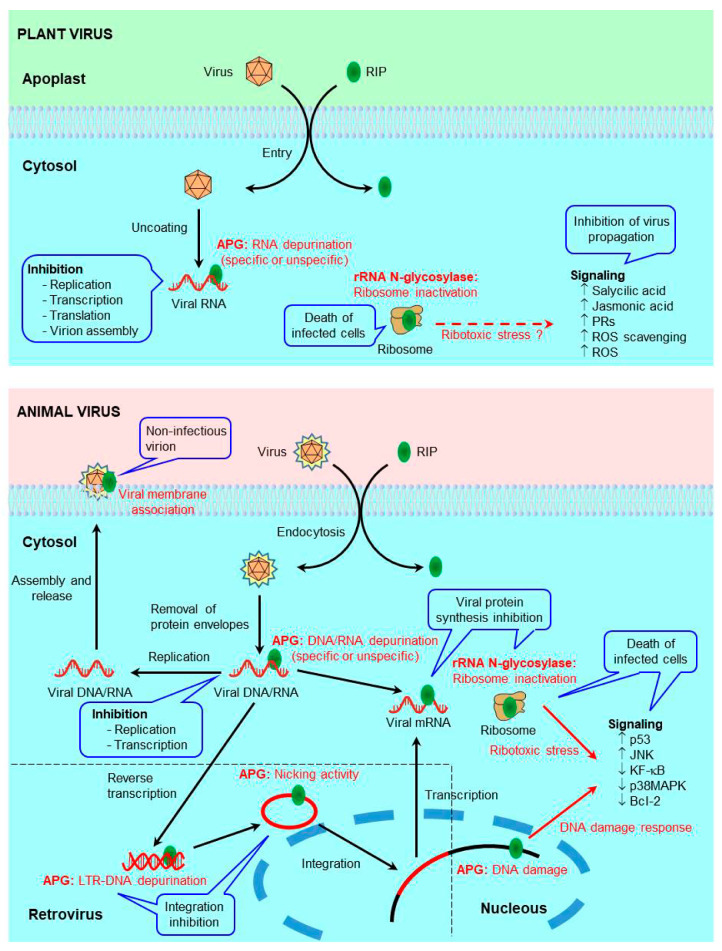
Proposed mechanisms for the antiviral activity of RIPs against plant viruses (upper panel), animal viruses (lower panel), and retroviruses (lower panel including dashed square). (**upper panel**) In plants, viral infection promotes the passage of the RIP from the apoplast to the cytosol. In the cytosol, it can inactivate ribosomes (rRNA glycosylase activity), causing the death of infected cells and thus preventing the spread of the virus. The RIP can also depurinate the viral RNA (adenine polynucleotide glycosylase, APG, activity), inhibiting its replication, transcription, translation, and assembly. It can also trigger antiviral defense signaling pathways, causing an increase in the levels of salicylic acid, jasmonic acid, pathogenesis-related (PR) proteins, and both reactive oxygen species (ROS) and ROS scavenging enzymes. (**lower panel**) In animal cells, the RIP can enter by pinocytosis or receptor-mediated endocytosis. RIP can inactivate ribosomes (rRNA glycosylase activity), causing the death of infected cells or inactivate the viral genome, DNA, or RNA (APG activity), preventing their replication, transcription, and translation. Some RIPs depurinate specific sequences (APG activity), blocking critical functions for the virus life cycle. In the case of retroviruses, the RIP can also depurinate the long terminal repeats (LTRs) (APG activity) or cleave the circular DNA (APG activity) preventing its integration into the cell genome. It can also be introduced into virions during budding (viral membrane association), making them less infective. Ribotoxic stress (rRNA glycosylase activity or APG activity on mRNA) and DNA damage (APG activity) caused by RIPs can trigger the activation of signaling pathways that cause infected-cell death preventing virus spreading.

RIPs have long been recognized as antiviral proteins in both plants and animals, but the mechanism responsible for this activity continues to be the subject of intense research today. The mechanism that triggers protection against viruses could have both common and different elements in plants and animals (Figure 1).

### 4.1. Antiviral Mechanisms of RIPs in Plants

#### 4.1.1. Protein Synthesis Inhibition (rRNA N-glycosylase)

It has long been known that RIPs can inhibit protein synthesis in plants [119,120,121,122]. The mechanism is the same as that described for inhibition of protein synthesis in animals, i.e., RIPs act as N-glycosylases of the major rRNA by removing a specific adenine from the sarcin-ricin loop (SRL), which is highly conserved in animals and plants [120]. Moreover, it has been shown that some RIPs can inhibit protein synthesis carried out by ribosomes of the same plants that produce them [123] and in addition, in the case of some RIPs, a positive correlation between rRNA N-glycosylase activity on tobacco ribosomes and antiviral activity against TMV has been reported [124].

The fact that RIPs do not cause cell death in the absence of the virus and allow plant growth is due to the fact that, at least for type 1 RIPs from dicots, they are synthesized as preproteins with a leader peptide that directs them into the apoplastic space [125]. Viral infection is supposed to facilitate the entry of the RIP, which inactivates cell ribosomes, causing cell death and preventing the virus from using the cellular machinery to replicate and spread [125]. So far, the mechanism by which the virus facilitates the entry of RIPs has not been shown, although the ability of viruses to modify plasma membrane permeability is well-known [126].

#### 4.1.2. Adenine Polynucleotide Glycosylase Activity

However, although some type 1 RIPs can inactivate ribosomes of some plants, they do not do so with those of others and usually act at much higher concentrations than in animal ribosomes [127]. In addition, mutants have been obtained from PAP that do not depurinate tobacco or reticulocyte lysate ribosomes but inhibit translation of brome mosaic virus (BMV) and potato virus X (PVX) [128].

The specificity of RIPs is highly variable, therefore some RIPs can act on other adenines in both animal [14] and plant [120,129] ribosomes. In addition, all RIPs release adenines from eukaryotic DNA and many of them also release adenines from other RNAs, including viral RNAs [15,22,87]. It has also been reported that some RIPs may have DNA nicking, DNase or RNase activities (Table 1). This can alter the life cycle of the virus, both its replication and transcription [130], translation [91], and assembly [131].

The adenine polynucleotide glycosylase activity on viral RNAs might be more specific. Thus, it has been reported that some RIPs can inhibit the translation of capped RNA by binding to the cap of viral RNAs and depurinating these RNAs downstream of the cap structure. For these RIPs, viral RNA depurination could be the main mechanism of their antiviral activity [51]. On the other hand, one of them (PAP) can also bind to translation initiation factors, allowing it to depurinate preferentially uncapped viral RNAs [103]. Viral capped RNA sequestration has also been proposed as an antiviral mechanism for MbRIP-1, a RIP from *Momordica balsamina* [132]. All this suggests that the antiviral mechanism of RIPs could be more complex than a simple and direct depurination of viral RNA.

#### 4.1.3. Antiviral Protection through Signaling Pathways

The other proposed mechanism involves signaling molecules that defend the plant from viral infection. However, different results have been obtained depending on the RIP studied and the approach used. Thus, it has been reported that α-momorcharin (α-MMC), in *N. benthamiana* plants sprayed with a solution of the RIP, up-regulates the expression of reactive oxygen species (ROS) scavenging-related genes, modulating ROS homeostasis and conferring resistance to TMV, ChiVMV, and CMV infection [81,133]. Additionally, this RIP also up-regulates some salicylic acid-responsive defence-related genes [81]. By contrast, the same RIP sprayed in *M. charantia* plants increases plant resistance to CMV but by increasing jasmonic acid biosynthesis and inducing ROS without a relevant increase in salicylic acid [82]. It has also been reported that α-momorcharin induces an increase of both jasmonic acid and salicylic acid in tobacco plants, enhancing TMV resistance [118]. On the other hand, it has been postulated that PAP generates a signal that leads to the overexpression of pathogenesis-related proteins rendering transgenic tobacco plants resistant to virus infection in the absence of an increase in the salicylic acid levels [129,134,135]. Finally, it has been reported that the expression of IRAb and IRIP in transgenic tobacco plants provides a strong local protection against TMV and TEV but without induction of pathogenesis-related proteins [77]. The relationship between the enzymatic activity of RIPs and their ability to induce production of signaling molecules in plants has not been studied. In animals, the enzymes that exert their cytotoxic function through modification of the sarcin-ricin loop (SRL), such as ricin, α-sarcin, or Shiga toxin, strongly activate signaling pathways through the mitogen-activated protein kinases (MAPKs) p38 and JNK [136]. The trichothecenes deoxynivalenol (DON) and T-2 toxin inhibit protein synthesis and have been shown to induce activation of ERK1/2 and p38 MAP kinase in several animal and human cell lines followed by increased cytokine production [137]. This ribosome mediated activation of MAPKs is termed ‘ribotoxic stress response’ [137]. In Arabidopsis, DON and T-2 toxin led to the expression of MPK3 and MPK6 MAP kinases, implicated as positive regulators of the hypersensitive response via ethylene signaling and ROS [137]. Therefore, it would be possible that the generation of signaling compounds by plants was a response to ribotoxic stress produced by RIPs.

### 4.2. Antiviral Mechanisms of RIPs in Animals

#### 4.2.1. Protein Synthesis Inhibition (rRNA N-glycosylase)

Early studies on the mechanism of antiviral action of RIPs in animal cells focused on their ability to inhibit protein synthesis [30]. Several type 1 RIPs (gelonin, *Momordica charantia* inhibitor, dianthin 32, and PAP-S) reduced viral production and plaque formation in HEp-2 cells infected with Herpes simplex virus-1 (HSV-1) or poliovirus I. In addition, the four RIPs inhibited protein synthesis more efficiently in cells infected with one of the two viruses than in uninfected cells, suggesting that RIPs inhibited viral replication by inhibiting protein synthesis of infected cells, presumably because they entered infected cells more easily than uninfected cells [30]. Although the mechanism by which viruses can facilitate the entry of RIPs is not established, it is known that type 1 RIPs can enter cells through pinocytosis or receptor-mediated endocytosis [138,139] and that both processes are stimulated by viruses [140,141].

#### 4.2.2. Adenine Polynucleotide Glycosylase Activity

However, RIPs can inhibit virus replication without apparently inactivating ribosomes [34,52,142,143]. The adenine polynucleotide glycosylase activity on viral RNA [57] or DNA [33] is able to inactivate the viral genome and explains inhibition of virus replication [37,142,143]. In addition, RIPs can also depurinate viral mRNAs, thus avoiding the synthesis of proteins that are vital for its functions [52,144,145]. In the case of HIV, a strong inhibition of the integration of viral DNA into the host genome [32,45,50], caused by the adenine polynucleotide glycosylase activity on LTRs (long-terminal repeats) [33,146,147] and the nicking activity on the supercoiled DNA [148,149] of the virus, has been reported. Trichosanthin is also able to enter viral particles during budding, resulting in virions unable to infect other cells [150,151].

#### 4.2.3. Antiviral Protection through Signaling Pathways

Finally, it has also been proposed that the antiviral activity of RIPs can be carried out through signaling pathways. Thus, it has been reported that RIPs promote p53 and c-Jun N-terminal kinase (JNK) activity [152,153] and block the activation of KF-κB, p38MAPK, and Bcl-2 [152,154,155] during viral infection. The modulation of these pathways would lead to the death of infected cells, thus preventing the spread of the virus. Cell DNA damage [152] or ribotoxic stress [153] caused by RIPs could trigger some of these signaling pathways. Ribotoxic stress response (RSR) is a response of cells to a variety of agents that affect the functions of ribosome, such as some antibiotics, alkaloids, mycotoxins, RIPs, ribotoxins, or ultraviolet radiation [136]. Ribotoxic stress is sensed by the MAP3K ZAKα that transduces the signal from ribosomes to activate MAP2K that in turn activates SAPKs. There are two SAPKs (stress-activated protein kinases) families in mammals: p38 and c-Jun N-terminal kinase (JNK). Activation of p38 induces cell-cycle arrest whereas activation of JNK promotes apoptosis [156], inducing both pro-survival and pro-apoptotic signaling. Additionally, mRNA damage by the adenine polynucleotide glycosylase activity of RIPs could trigger RSR as has been reported for ultraviolet radiation [156]. However, much research is still required to clarify how RIPs protect cells from viral infection through these pathways.

Therefore, RIPs can exert their antiviral effect through different mechanisms that could originate from their activity on the different nucleic acids from both the virus and the infected cell. Depending on the type of RIP, virus and infected cell, some mechanisms could predominate over others and more research is required to determine in each case which are the predominant ones.

## 5. Experimental Therapy

Because of its strong antiviral activity, RIPs have been used in experimental therapy, especially to treat the acquired immune deficiency syndrome (AIDS), but also against hepatitis, chikungunya, dengue, and lymphomas caused by the Epstein–Barr virus. Additionally, they have also been tested in vivo against viruses that infect animals, such as the murine cytomegalovirus, the Pichinde virus, or the simian–human immunodeficiency virus (Table 4).

### 5.1. RIPs and PEGylated RIPs

Trichosanthin (GLQ223) was used alone [61,157] or in combination with zidovudine (azidothymidine, AZT) [158] in clinical trials with AIDS patients. Trichosanthin infusions were safe and relatively well tolerated [157]. In patients, a decrease in serum p24 antigen [61] and an increase in CD4^+^ and CD8^+^ T cells [157,158] were observed. Recently, it has also been reported that maize RIP reduces the viral load of an HIV-related virus, the simian–human immunodeficiency virus in Chinese rhesus macaques [27].

Despite its potential as therapeutic agents, the strong immunogenicity, allergic reaction, and short half-life are the biggest barriers to their application as therapeutic agents. Polyethylene glycol (PEG) conjugation (PEGylation) can confer on these proteins, increasing plasma half-life, decreasing toxicity, and reducing immunogenicity and antigenicity. PEGylated alpha-momorcharin and MAP30 showed about 60%–70% antivirus activities against HSV-1, and at the same time decreased 50%–70% immunogenicity when compared with the non-PEGylated proteins [40].

### 5.2. Immunotoxins and Other Conjugates

RIPs have been used in medicine mainly as the toxic part of immunotoxins, that is, chimeric proteins consisting of an antibody specifically directed against a target, linked to a toxin of plant or bacterial origin. The design of immunotoxins has been improved over the past 40 years to minimize the off-target toxicity and immunogenicity [159,160]. Several types of antiviral immunotoxins have been constructed using either bacterial toxins (or their fragments) such as pseudomonas exotoxin A or diphteria toxin [161], and RIPs from plants (Table 4). The most commonly used RIP has been the ricin A-chain and the most studied virus the HIV. Viral proteins (gp41, gp120, or gp 160) or proteins from infected cells (CD4, CD25, or CD45RO) have been selected as targets. Despite the success of highly active antiretroviral therapy (HAART), antiviral immunotoxins continue to be developed in order to deplete persisting HIV-infected cell reservoirs [162]. Immunotoxins have also shown to be active in vitro against Epstein–Barr [163,164] and Pichinde [31] viruses and in vivo (in combination with the synthetic analogue of 2′-deoxy-guanosine ganciclovir) against the murine cytomegalovirus [165].

Targeting can also be carried out by conjugating RIPs with other proteins or peptides that specifically bound to viral proteins or proteins present only in infected cells [49,166].

### 5.3. Designed Antiviral Proteins and Nanocapsules

RIPs have also been used to design antiviral proteins. One of these engineered proteins contains an internal sequence that is recognized by the HIV protease and that is blocking the N-glycosylase activity of the RIP. This protein is activated in infected cells and has shown antiviral activity [28]. Similarly, variants of the ricin A-chain with the sequence recognized by the HIV protease in the C-terminus are activated in infected cells and show antiviral activity [29].

Another approach is to fuse the sequences of RIPs with antimicrobial peptides such as latarcin, thanatin, protegrin-1, and plectasin that are able to inhibit viral replication inside the infected cells, viral entry and replication, dengue NS2B-NS3 serine protease, and virus replication, respectively [42,53]. The aim is to target different stages of the viral life cycle. Thus, the peptide-fusion proteins Latarcin-PAP1-Thanatin and Protegrin1-MAP30-Plectasin inhibit virus replication in vitro and protect the virus-infected mice from chikungunya and dengue viruses, respectively [42,53]. Another fusion protein containing ricin A-chain and PAP-S displays antiviral activity in vitro against hepatitis B virus suggesting a synergistic activity of both proteins [167]. This has encouraged its authors to propose it as an anti-SARS-CoV-2 agent [75].

The latest approach is the use of nanocapsules to deliver RIPs to virus-infected cells. Nanocapsules are vesicular objects in which the encapsulated compound is confined in an internal cavity surrounded by an outer membrane [182,183]. Nanocapsules containing MAP30 [180] or ricin A-chain [181] have shown antiviral activity in vitro against HIV. In the latter case, targeting has been achieved by using peptide crosslinkers that are sensitive to cleavage by HIV-1 protease [181].

### 5.4. Side Effects of RIP Therapy

Although trichosanthin was, in general, well tolerated in clinical trials when used in AIDS patients [157], some side effects were reported [61,157,158]. Clinical trials using RIPs as antivirals are scarce, but there are many clinical trials that have used RIPs as part of immunotoxins for the treatment of malignancies [9,64,184]. Side effects that may be mild or moderate like fever, nausea, vomiting, diarrhea, myalgia, edema, and hypoalbuminemia have been reported in these trials. Other effects are severe, such as immunogenicity and vascular leak syndrome (VLS), and could limit the therapeutic use of immunotoxins [64,184]. Immunogenicity may be the result of the formation of human anti-mouse antibodies (HAMA) or human anti-toxin antibodies (HATA). These antibodies can prevent repeated treatment cycles. The development of immunotoxins containing humanized antibodies or the use of part of antibodies containing only the variable domains can solve this problem [64,184]. To address the problem of the immunogenicity of RIPs, PEGylation [40,184] and elimination of epitopes through genetic manipulation have been used [184]. Vascular leak syndrome, characterized by increased vascular permeability, is caused by the nonspecific binding of RIP to vascular endothelial cells. The identification and elimination of some peptides present in RIPs, nonessentials for RIP activity and responsible for this unspecific binding, have allowed the obtaining of less toxic recombinant RIPs [184].

## 6. Genetically Engineered Virus-Resistant Plants

Viruses cause epidemics in all major crops, representing a significant restriction on the yield and quality of agricultural production. As strict intracellular pathogens, they cannot be chemically controlled and prophylactic measures consist mainly in the destruction of infected plants and biocide applications to limit the population of vector organisms (arthropods, nematodes, and plasmodiophorids). A powerful alternative often used in agriculture is based on the use of crop genetic resistances, an approach that depends on mechanisms governing plant-virus interactions [185]. Several transgenic plants carrying virus resistance genes have been obtained by transferring virus-derived genes, including viral coat proteins, replicases, movement proteins, defective interfering RNAs, non-coding RNA sequences and proteases into susceptible plants, or non-viral genes including R genes, microRNAs, RIPs, protease inhibitors, dsRNAses, RNA modifying enzymes, and scFvs [186]. In recent years, transgenic plants carrying RIP genes that are resistant to fungi, insects and, above all, viruses have been reported. Thus, transgenic plants bearing RIP genes have been obtained that are resistant to a wide variety of viruses (Table 5). 

Most of the times, tobacco has been transformed (*Nicotiana tabacum* L. and *N. benthamiana* Domin) but also potato (*Solanum tuberosum* L.) and tomato (*Lycopersicon esculentum* Mill.). *Agrobacterium tumefaciens* containing the plant transformation vectors has been used to transform either tobacco by the leaf disc co-cultivation method or potato (*S. tuberosum*) by the stem or tuber section co-cultivation method. The CaMV 35S promoter has always been used to express the RIPs, except in the case of dianthin 30 [84]. In the case of trichosanthin, tissue-specific promoters have also been used [80]. The CaMV 35S promoter is the most studied and most widely used plant promoter for transgenic expression [189], it is a very strong constitutive promoter that facilitates a high level of RNA transcription in a wide variety of plant species. For effective protection against viruses, it is preferable to achieve high levels of RIP expression since there is a direct correlation between expression level and resistance to viruses [78]. So, for example, in lines expressing small amounts of curcin 2, symptoms of TMV infection begin to appear after about 7 days, while lines that accumulate the highest level of curcin 2 (about 1.45 µg/mg) begin to develop symptoms after about 18 days.

Using the promoter CaMV 35S, plants with a RIP content of up to 2.7% of the total soluble protein have been obtained [80]. However, a high expression of RIP results in plants with an aberrant phenotype, which usually includes leaf mottling, extreme leaf discoloration, stunted leaf growth and/or excessive curvature, slow rooting and growth rates, and high plant mortality rates [80,188]. This could be because some RIPs can kill plant cells by inactivating their ribosomes [120,121,122]. Several approaches have been used to overcome this problem. One strategy might be to introduce the gene encoding for the preprotein [80], this allows the RIP to accumulate in the apoplasma instead of the cytosol, thus preventing access to the ribosomes. Transgenic tobacco plants expressing the preprotein of trichosanthin exhibited resistance to cucumber mosaic virus (CMV) and tobacco mosaic virus (TMV) but did not show an abnormal phenotype [80]. In the case of PAP, despite being the most widely used, it inhibits protein synthesis and is toxic to plant cells, but transgenic plants have been obtained with mutants that are not toxic to the plant maintaining the antiviral activity [188]. The lack of toxicity of these mutants has been attributed to a change in the location of the protein preventing contact with ribosomes [188]. PAP (PAPI) has also been replaced by PAPII in order to obtain virus-resistant plants [104]. The protein sequence of PAPII shows only 41% identity to PAPI. PAPII expressed in transgenic tobacco was correctly processed to the mature form and accumulated to at least 10-fold higher levels than wild-type PAP (up to 250 ng/mg PAPII). PAPII is less toxic than PAP and symptomless transgenic lines expressing PAPII were resistant to TMV and PVX [104]. Another approach is to use a promoter that is induced by viral infection, thus, the gene that encodes for dianthin 30 was introduced into *N. benthamiana* and expressed from the promoter ACMV virion-sense [84]. This promoter is induced specifically by the ACMV infection and transgenic plants displayed a normal phenotype and were resistant to ACMV [84].

Finally, it should be noted that some virus-resistant transgenic plants have been reported to be also resistant to fungi [78,104], which adds interest to this type of approach to improve crop resistance.

## 7. Conclusions

After decades of research, RIPs continue to be a topic of interest and a useful tool in many research fields. The new advances in plant molecular biology, virology, immunotherapy, and nanotechnology open new possibilities in the use of RIPs in medicine and agriculture in order to find solutions to the continuous challenge posed by viruses to human health and crop yields.

## Figures and Tables

**Table 1:** Proposed activities and other biological properties of ribosome-inactivating proteins (RIPs).

Activity	Example of RIP	References
Agglutinin	Ricin	[8]
Antiviral	PAP	[10]
rRNA N-glycosylase	Ricin	[11]
Adenine polynucleotide glycosylase	Saporin-L1	[15]
rRNA N-glycosylase/lyase	Gypsophilin/RALyase	[16]
RNase	BBAP1	[17]
DNase	BBAP1	[17]
Phosphatase	Trichosanthin	[18]
Superoxide dismutase	Camphorin	[19]
Phospholipase	Ricin	[20]
Chitinase	TKC 28-I	[21]
DNA nicking	BE27	[22]
Apoptosis induction	Stenodactylin	[4,23]
Necroptosis induction	Stenodactylin	[4,23]
Autophagia induction	Abrus Agglutinin	[24]
Senescence induction	JIP60	[25]
Plant tissue necrosis	JIP60	[26]

**Table 2:** RIPs active against animal viruses. RIPs with antiviral activity, the families and species from which they have been obtained and the viruses in which this activity has been demonstrated are shown.

Species and RIP	Virus	References
POACEAE		
*Zea mays* L.		
Maize RIP	HIV, SHIV	[27,28]
EUPHORBIACEAE		
*Ricinus communis* L.		
Ricin A chain	HIV	[29]
*Suregada multiflora* (A.Juss.) Baill. (=*Gelonium multiflorum* A.Juss.)		
Gelonin	HIV, HPV, HSV, PICV,	[2,30,31,32]
GAP31	HIV	[33,34]
CUCURBITACEAE		
*Trichosanthes kirilowii* Maxim		
Trichosanthin (TCS)	HBV, HIV, HSV	[32,35,36,37,38]
TAP29	HIV	[36]
Trichobitacin	HIV	[36,39]
*Momordica charantia* L.		
Momordin (*M. charantia* inhibitor)	HPV, HSV	[30]
Alpha-momorcharin (α-MMC)	HBV, HIV, HSV	[2,32,40,41]
Beta-momorcharin	HIV	[2,32]
Momordica antiviral protein (MAP30)	DENV-2, HHV8, HBV, HIV, HSV	[35,42,43,44,45,46]
*Momordica balsamina* L.		
Balsamin	HIV	[47]
*Luffa cylindrica* (L.) M.Roem.		
Luffin	HIV	[32]
*Bryonia cretica* subsp. *dioica* (Jacq.) Tutin (=*Bryonia dioica* Jacq.)		
Bryodin	HIV	[48]
CARYOPHYLLACEAE		
*Saponaria officinalis* L.		
Saporin	HIV	[32,49,50]
*Dianthus caryophyllus* L.		
Dianthin 32 (DAP32)	HIV, HPV, HSV	[30,34]
Dianthin 30 (DAP30)	HIV	[34]
*Agrostemma githago* L.		
Agrostin	HIV	[2,32]
PHYTOLACCACEAE		
*Phytolacca americana* L.		
PAP (PAPI)	CHIKV, FLUV, HBV, HIV, HPV,	[10,35,51,52,53,54,55,56,57]
	HSV, HTLV, JEV, LCMV	
PAPII	HIV	[57]
PAPIII	HIV	[57]
PAP-S	HSV, HPV, HBV	[30,56]

Virus name abbreviations: CHIKV (chikungunya virus), DENV (dengue virus), FLUV (human influenza virus), HBV (hepatitis B virus), HHV (human gammaherpesvirus), HIV (human immunodeficiency virus), HPV (human poliovirus), HSV (herpes simplex virus), HTLV (human T-cell leukemia virus), JEV (Japanese encephalitis virus), LCMV (lymphocytic choriomeningitis virus), PICV (Pichinde virus), SHIV (simian–human immunodeficiency virus).

**Table 3:** RIPs active against plant viruses. RIPs with antiviral activity, the families and species from which they have been obtained and the viruses in which this activity has been demonstrated are shown.

Species and RIP	Virus	References
IRIDACEAE		
*Iris x hollandica* Tub.		
IRIP	TMV, TEV	[77]
IRAb	TMV, TEV	[77]
EUPHORBIACEAE		
*Jatropha curcas* L.		
Curcin 2	TMV	[78]
CUCURBITACEAE		
*Trichosanthes kirilowii* Maxim		
Trichosanthin	TuMV, CMV, TMV	[79,80]
*Momordica charantia* L.		
α-Momorcharin	CMV, ChiVMV, TMV, TuMV	[81,82]
LEGUMINOSAE		
*Senna occidentalis* (L.) Link (=*Cassia occidentalis* L.)		
Cassin	TMV	[83]
CARYOPHYLLACEAE		
*Saponaria officinalis* L.		
Saporin	BMV, TMV, AMV	[51]
*Dianthus caryophyllus* L.		
Dianthin 30	ACMV, TMV	[84,85]
Dianthin 32	TMV	[85]
AMARANTHACEAE		
*Beta vulgaris* L.		
BE27	TMV, AMCV	[86,87]
*Amaranthus tricolor* L.		
AAP-27	SHMV	[88]
*Amaranthus viridis* L.		
Amaranthin	TMV	[89]
*Celosia argentea* L. (=*Celosia cristata* L., = *Celosia plumosa* (Voss) Burv.)		
CCP 25	BMV, PMV, TMV, SHMV, ICRSV	[90,91,92]
CCP 27	TMV, SHMV, ICRSV	[92,93]
*Chenopodium album* L.		
CAP-I	TMV, SHMV	[94]
CAP-II	TMV, SHMV	[94]
CAP30	TMV	[95]
*Salsola longifolia* Forssk.		
SLP-32	BYMV, TNV	[96]
*Spinacia oleracea* L.		
VI (SoRIP2)	TMV	[97,98]
PHYTOLACCACEAE		
*Phytolacca insularis* Nakai		
PIP	TMV, CMV, PVY, PVX, PLRV	[99]
*Phytolacca dioica* L.		
Dioicin 2	TMV	[87]
PD-S2	TMV	[87]
PD-L1	TNV	[100]
PD-L4	TMV, TNV	[87,100]
*Phytolacca americana* L.		
PAP (PAPI)	BMV, TMV, AMV, TBSV, SPMV, ZYMV	[51,58,101,102,103,104,105]
	CMV, PVY, PVX, TEV, SBMV	
PAPII	TMV, PVX	[104]
PAP-S	AMCV	[105]
NYCTAGINACEAE		
*Boerhaavia diffusa* L.		
BDP-30	TMV	[106]
*Mirabilis expansa* (Ruiz & Pav.) Standl.		
ME1	TMV, BMV	[51]
*Mirabilis jalapa* L.		
MAP	TMV	[107]
*Bougainvillea spectabilis* Willd.		
Bouganin	ZYMV, AMCV	[105,108]
*Bougainvillea buttiana* Holttum & Standl.		
BBAP1	SHMV	[17]
BBP-24	TMV, SHMV	[109,110]
BBP-28	TMV, SHMV	[109,110]
BASELLACEAE		
*Basella alba* L. (=*Basella rubra* L.)		
RIP2	AMCV	[105]
LAMIACEAE		
*Volkameria inermis* L. (=*Clerodendrum inerme* (L.) Gaertn.)		
CIP-29	TMV, PRSV, SHMV	[111,112]
*Volkameria aculeata* L. (=*Clerodendrum aculeatum* (L.) Schltdl.)		
CA-SRI (CAP-34)	TMV, SHMV, PRSV	[113,114,115]
ADOXACEAE		
*Sambucus nigra* L.		
SNAI’	TMV	[116]
Nigrin b (SNAV)	TMV	[76]

Virus name abbreviations: ACMV (African cassava mosaic virus), AMCV (artichoke mottled crinkle virus), AMV (alfalfa mosaic virus), BMV (brome mosaic virus), BYMV (bean yellow mosaic virus), ChiVMV (Chilli veinal mottle virus), CMV (cucumber mosaic virus), ICRSV (Indian citrus ringspot virus = citrus ringspot virus, CRSV), PLRV (potato leafroll virus), PMV (pokeweed mosaic virus), PRSV (papaya ringspot virus), PVX (potato virus X), PVY (potato virus Y), SBMV (southern bean mosaic virus), SHMV (sunn-hemp mosaic virus = sunn-hemp rosette virus, SRV), SPMV (satellite panicum mosaic virus), TBSV (tomato bushy stunt virus), TEV (tobacco etch virus), TMV (tobacco mosaic virus), TNV (tobacco necrosis virus), TuMV (turnip mosaic virus), ZYMV (zucchini yellow mosaic virus).

**Table 4:** Ribosome-inactivating proteins used in experimental antiviral therapy. RIPs have been used alone, PEGylated, or as part of immunotoxins, conjugates, engineered proteins, or nanocapsules.

Virus	Target	RIP	References
**RIPs alone**			
HIV	HIV infected cells	TCS	[61,157,158]
SHIV	SHIV infected cells	Maize RIP	[27]
**PEGylated RIPs**			
HSV-1	HIV infected cells	α-MMC	[40]
	HIV infected cells	MAP30	[40]
**Immunotoxins**			
HIV	gp 120	RAC, PAP-S, PAC, Gelonin	[168,169,170,171,172]
	gp 41	RAC, PAC, Gelonin	[170,171,172,173,174,175]
	gp 160	RAC	[173]
	CD45RO	RAC	[176]
	CD4	PAP	[143,177]
	CD25	RAC	[178]
PICV	PICV	Gelonin	[31]
EBV	CD30	Saporin 6	[163]
	EBV/C3d receptor	Gelonin	[164]
MCMV	MCMV	RAC	[165,179]
**Conjugates**			
HIV	gp 120	RAC	[166]
	CD8^+^ T-cells	Saporin	[49]
**Engineered proteins**			
HIV	HIV protease	RAC	[29]
	HIV protease	Maize RIP	[28]
CHIKV	Viral life cycle	PAP	[53]
DENV	Viral life cycle	MAP30	[42]
HBV	HBV infected cells	RAC-PAP	[167]
**Nanocapsules**			
HIV	HIV infected cells	MAP30	[180]
	HIV protease	RAC	[181]

Virus name abbreviations: CHIKV (chikungunya virus), DENV (dengue virus), EBV (Epstein–Barr virus), HBV (hepatitis B virus), HIV (human immunodeficiency virus), HSV (herpes simplex virus), MCMV (murine cytomegalovirus), PICV (Pichinde virus), SHIV (simian–human immunodeficiency virus). RIP name abbreviations: MAP (Momordica antiviral protein), α-MMC (alpha-momorcharin), PAC (Pulchellin A-chain), PAP (pokeweed antiviral protein), RAC (ricin A-chain), TCS (trichosanthin).

**Table 5:** Transgenic plants bearing RIP genes. The degree of protection achieved is indicated as the percentage reduction of lesions, infected plants or detected virus levels, or as the delay in the onset of symptoms.

RIP	Host	Virus	Protection	Ref.
IRIP	*Nicotiana tabacum*	TMV, TEV	73% L.L.	[77]
IRAb	*Nicotiana tabacum*	TMV, TEV	54% L.L.	[77]
Curcin 2	*Nicotiana tabacum*	TMV	9 D.D.	[78]
Trichosanthin	*Nicotiana tabacum*	TuMV	100% L.L.	[79]
	*Nicotiana tabacum*	TMV, CMV	14 D.D.	[80]
	*Lycopersicon esculentum*	TMV, CMV	100% L.I.P.	[187]
Cassin	*Nicotiana tabacum*	TMV	13 D.D.	[83]
Dianthin 30	*Nicotiana benthamiana*	ACMV	100% L.L.	[84]
PIP	*Solanum tuberosum*	PVY, PYX, PLRV	98% L.V.L	[99]
PAP	*Nicotiana tabacum*	PVY, PYX, CMV	100% L.I.P.	[102,188]
	*Nicotiana benthamiana*	PVY	67% L.I.P.	[102]
	*Solanum tuberosum*	PVY, PYX	84% L.I.P.	[102]
PAPII	*Nicotiana tabacum*	TMV, PVX	89% L.L.	[104]
SNAI’	*Nicotiana tabacum*	TMV	59% L.L.	[116]
Nigrin b (SNAV)	*Nicotiana tabacum*	TMV	43% L.L.	[76]

Virus name abbreviations: ACMV (African cassava mosaic virus), CMV (cucumber mosaic virus), PLRV (potato leafroll virus), PVX (potato virus X), PVY (potato virus Y), TEV (tobacco etch virus), TMV (tobacco mosaic virus), TuMV (turnip mosaic virus). Protection abbreviations: L.L. (less lesions), D.D. (days of delay), L.I.P. (less infected plants), L.V.L. (less virus level).

## Data Availability

Data are available upon request. Please, contact the contributing authors.
